# Formulation Design of Orally Disintegrating Film Using Two Cellulose Derivatives as a Blend Polymer

**DOI:** 10.3390/pharmaceutics17010084

**Published:** 2025-01-10

**Authors:** Yoshiko Takeuchi, Fumika Hayakawa, Hirofumi Takeuchi

**Affiliations:** Laboratory of Advanced Pharmaceutical Process Engineering, Gifu Pharmaceutical University, 5-6-1 Mitahora-Higashi, Gifu 502-8585, Japan

**Keywords:** orally disintegrating film, blend of polymers, hydroxypropyl cellulose, hydroxypropyl methylcellulose, FTIR, DSC

## Abstract

**Background/Objectives**: Orally disintegrating film (ODF) is prepared using water-soluble polymers as film-forming agents. To improve mechanical and disintegration properties, some polymers need to be blended with others. This study aimed to investigate the utility of hydroxypropyl cellulose (HPC) and hydroxypropyl methyl cellulose (HPMC) as blend film-forming components for ODFs. **Methods**: Placebo ODFs were prepared using polymer mixtures with blend ratios ranging from 20% to 80% HPC with HPMC. Mechanical properties, including tensile strength, elastic modulus, elongation at break, and folding endurance, as well as disintegration times, were evaluated. Additionally, blend films incorporating donepezil hydrochloride (DH) as a model active pharmaceutical ingredient (API) were prepared and assessed to determine their mechanical properties and disintegration behavior. **Results**: Blend films were successfully formed using HPMC/HPC solutions. The 40/60 and 20/80 HPMC/HPC blends exhibited the lowest mechanical strength and elongation, whereas blends containing more than 40% HPC demonstrated shorter disintegration times. Films with DH were successfully formed, though the addition of DH reduced tensile strength and elongation. The decline in mechanical properties was mitigated in HPMC/HPC blend films. Our results, including DSC and FTIR results, led us to conclude that the HPMC/HPC blend films were micro-immiscible, but they were macro-miscible when the amount of the minor component was sufficiently small. **Conclusions**: HPMC/HPC blends in appropriate ratios are effective as film-forming polymers for ODFs. The addition of DH impacts the mechanical properties, but the decline is less pronounced when using HPMC/HPC blends.

## 1. Introduction

Orally disintegrating films (ODFs) are among the most preferred drug dosage forms for pediatric patients [1,2] and geriatric patients [3]. They are produced using various methods, such as solvent casting, electrospinning, hot-melt extrusion, and 2D- or 3D- printing [4], to create either a single-film sheet or a multilayered sheet. ODFs must disintegrate rapidly in the oral cavity and be sufficiently strong for manufacturing and administration [2,5]. Water-soluble polymers serve as film-forming agents, while formulations incorporate various components such as plasticizers, surfactants, and fillers. Among these components, film-forming polymers primarily dominate the properties of ODFs, making appropriate polymers crucial. Indeed, there are many to choose from. Various natural, synthetic, and semi-synthetic polymers have been used in ODF investigations and manufacturing processes [6,7].

In pharmaceutical applications, polymers are often blended to improve and combine their properties without altering their structures and functions [8]. In the formulation design of fast-dissolving ODF strips, combinations of different polymers, or of the same polymers with different molecular masses, are often used as film formers to obtain their desired properties [9]. Kalkarni et al. [10] explored the film-forming capacities, appearances, and disintegration times of different film-forming polymers and blends of them as film-forming agents for fast-dissolving ODF strips. Pacheco et al. [11] reviewed ODFs prepared from natural polymers, such as pullulan, maltodextrin, and others, and showed that some natural polymers need to be blended with synthetic, semisynthetic, or other natural polymers to achieve better mechanical and disintegration properties. Visser et al. [12] examined different combinations of film-forming agents for orodispersible films and found that the best ratios depended on the polymers being blended. Elbadawi et al. [13] investigated a 50/50 blend of these two polymers for oral films using the micro-syringe 3D-printing method and found many defects on the surfaces of the films’ fibrous structures. They concluded that the incompatibility of these two polymers led to the uneven mixing of the film fibers. Ding et al. [14] sought to improve the known flaws of collagen films by blending collagen with HPMC, recognized for its widespread availability, superior film-forming capacity, and good biocompatibility and biodegradability. Blending improved the film properties because of hydrogen interactions and entanglement between collagen and HPMC molecules, resulting in good miscibility. El-Malah and Nazzal [15] investigated a three-way blend film of pullulan, HPMC, and PVP to optimize a fast-dissolving film containing sodium diclofenac. High concentrations of pullulan were deemed essential for fast disintegration, while the addition of HPMC and PVP was found to modulate the mechanical strength of the films. Considering all these valuable reports, it becomes clear that blending multiple polymers can significantly enhance ODF properties. Both the components and the ratios of these polymers must be optimized for ODF preparations.

The aim of this study was to investigate the feasibility of an HPMC/HPC blend as a film-forming polymer for ODFs. These two polymers were chosen because they are similar types of cellulose derivatives and are frequently used as film formers in pharmaceutical processes due to their film-forming capabilities and easier disintegration [16,17,18]. In their investigation of mucoadhesive films with lidocaine, Repka et al. [19] compared pure HPC with HPC/HPMC (80:20) formulations using the hot-melt extrusion technique. The incorporation of HPMC into HPC formulations increased the bioadhesive strength of films and retarded drug release. In the preparation of orodispersible films for individualized pharmacotherapy, Visser et al. [12] tested various film-forming agents blended to evaluate the prepared films. Among them, HPMC/HPC (80:20) with 20% glycerol films showed good results, including moderate high tensile strength, low Young’s modulus, and high elongation at break, indicating their potential as blending film formers for ODFs. First, we investigated the properties of ODFs using blends with HPMC and HPC at several ratios as film-forming agents. We then researched the effects of blending HPC in the film formulations on the morphological, mechanical, physical, and disintegration properties of the films. The findings revealed that several combinations of these polymers can function as high-performance film-forming blends for ODFs. We focused on the blend solutions with HPMC and HPC polymers. The placebo films prepared using these solutions were evaluated with FTIR and DSC to clarify the film structures, opening the effect on their mechanical properties. Second, we used donepezil hydrochloride (DH) as an API for ODFs. The suitability of polymers and polymer blends as film-forming agents for DH films was investigated. ODFs with DH using three types of film-forming agents—HPMC, HPMC/HPC blend, and HPC—were compared.

## 2. Materials and Methods

### 2.1. Materials

Hydroxypropyl methylcellulose (HPMC; TC-5R; MW of about 35,600 and viscosity of 6 mPa·s) was kindly provided by Shin-Etsu (Tokyo, Japan), and hydroxypropyl cellulose (HPC; HPC-SL; MW of ~100,000 and viscosity of 3–5.9 mPa·s) was kindly provided by Nippon Soda (Tokyo, Japan). Ethanol (Nacalai Tesque, Kyoto, Japan) used was reagent-grade material.

### 2.2. Methods

#### 2.2.1. Film Preparation

The solution/solvent casting method [20,21] was adopted for film formation. The water-soluble polymers, HPMC and HPC, served as film formers. The formulations, along with the ratios of the two polymers and APIs, are detailed in Table 1. The solutions/dispersions were prepared, and the films were cast at room temperature. The procedures can be described in brief as follows:

(a) HPMC was dispersed in ethanol with stirring, and then, a specified amount of distilled water was added. HPC was dissolved carefully and gradually by mixing ethanol and distilled water. To prepare the blend solution, HPMC and HPC were mixed and dispersed in ethanol, and then, distilled water was added. The solvents were mixed at an ethanol/distilled water ratio of 2:1 (*w/w*).
(b) If necessary, the solutions were mixed with API with sufficient stirring.
(c) Before casting, the film solutions/dispersions were degassed using a desiccator and a vacuum pump (ULVAC kiko, Yokohama, Japan) and kept here for more than 24 h. The films were spread on a base film (polypropylene: Pylen^®^; Toyobo, Osaka, Japan), fixed onto a heat-resisting glass plate using a YBA-type baker applicator (Yoshimitsu Seiki, Tokyo, Japan).
(d) The films were kept in a dry chamber (KCV-4D; Advantec, Tokyo, Japan) with an air current to evaporate the solvent or water (40 °C) for more than 2 h. After drying, the defects on the surface of films were checked. They were then cut into 20 mm × 30 mm pieces, stored at room temperature for at least 24 h, and evaluated.
(e) Before casting, the viscosity of HPMC/HPC blend solutions was measured using a viscometer (TV-10; Tokisangyo, Tokyo, Japan). For this measurement, the rotation speed was 60 rpm, and the type of rotor used was M2 with a guard, as described in previous studies [22]. The solutions were maintained in a water bath at 25 °C until the sample temperature reached the desired level. As shown in Table 1, the polymer (or polymer blend)/solvent ratios were 1:10 in the casting solution.

#### 2.2.2. Mechanical Properties

(a) Tensile Strength of ODFs

The mechanical property of tensile strength (TS) was measured using a creep meter (RE-3305S; Yamaden, Tokyo, Japan), as described in previous studies [20,21,22,23,24]. The 20 mm × 30 mm film strip pieces were vertically secured with two grips 17 mm apart (i.e., the initial separation distance), and then pulled at a constant rate of 0.5 mm/s. The maximum fracture force, the force reached just before the film strips ruptured, was recorded. The measurements were conducted in triplicate using three film samples for each formulation type. The TS (MPa), the elastic modulus (EM) (MPa), and the elongation at break (EB) (%) were computed during the tensile test.

(b) Folding Endurance (FE) of ODFs

The bending test equipment was a Desktop Model Endurance Test Machine (TCDM111LH, C2BR; Yuasa System, Okayama, Japan), and it was used as described in previous studies [23]. Briefly, a strip of the sample film was secured between the two blocks of the clamp, and a downward weight was applied. The clamp was positioned to pivot at a certain angle around the folding point, initiating repeated folding at the folding point, i.e., the tip of the folding clamp. The FE number was defined as the count of folds before a film strip ruptured. The defined conditions were operating angle of ±135°; operating speeds of 90 rpm; R-block (R) tip size of 0.38 mm; and a weight of 100 g (the 32 g clip weight was applied separately).

#### 2.2.3. Film Thickness

The film thickness was measured using a micrometer (Mitutoyo, Kawasaki, Japan) with an accuracy of 1 μm, as described previously [20,21,22,23,24]. Each sample film was measured at three different positions per strip, and the mean values were calculated.

#### 2.2.4. Disintegration Test

The disintegration time (DT) was evaluated with a disintegration test method developed for ODFs, detailed in a previous paper [24]. Tricorptester^®^ (Okada Seiko, Tokyo, Japan) was used as the disintegration test apparatus. In this method, a test medium was dropped from the tube onto the film strip under constant conditions, and an optical passage confirmation sensor was used to enable the automatic detection of the DT of the ODFs, after which, the DT was automatically recorded. The disintegration test medium was an artificial saliva solution maintained at 37 °C in an incubator. The dropping height and flow rate were 7 cm and 6 mL/min, respectively, and a non-trap continuous dropping method was used. All measurements were performed three times, and means and standard deviations (SDs) were used for analysis.

#### 2.2.5. Differential Scanning Calorimetry (DSC) Analysis

The thermal stability of the blend films was assessed using a differential scanning calorimetry (DSC) device (EXSTER DSC-6200, Seiko Instruments, Chiba, Japan). Each 5 mg sample was precisely weighed into a pan, sealed, and then scanned from 25 to 250 (°C) at a heating rate of 5 (°C/min) under a N_2_ atmosphere. The sampling resonance was 0.5 s.

#### 2.2.6. Fourier Transform Infrared Spectroscopy (FTIR) Analysis

The spectra of the blended films were recorded using an FTIR spectrophotometer (Frontier FTIR, PerkinElmer, Japan, Yokohama, Japan). The measurements were performed at a resolution of 1 cm^−1^ and within the range of 650–4000 cm^−1^.

#### 2.2.7. Morphological Observation

A scanning electron microscope (JSM-T330; JEOL, Tokyo, Japan) was used to observe the surfaces and cross-sections of the films. The cross-sectional samples were obtained after the tensile test.

### 2.3. Statistical Analysis

The results obtained from the physical evaluations were analyzed statistically using Student’s *t*-test. Probability values of *p* < 0.05 were considered to indicate statistically significant differences.

## 3. Results and Discussion

### 3.1. Characteristics of the HPMC/HPC Blend Film

In the manufacture of ODFs, blends of water-soluble polymers are often used as film formers to improve their morphological, mechanical, and disintegration properties and to enhance the dissolution and uniformity of APIs [10,11]. In this study, HPMC and HPC were chosen as blending components based on the results of several reports [16,17,25], including our own [20,22,23]. HPMC is a superior polymer for ODFs because of its ease of use, film-forming capacity, good biodegradability, and other factors [14]. HPC is also a usable polymer for ODFs due to its film-forming capacity, rapid disintegration, and other characteristics [20]. These two polymers have the same basic cellulosic backbone and other similarities in their chemical structures [8]. Visser et al. [12]. demonstrated the feasibility of a 2:1 mixture of HPMC and HPC in producing ODFs with moderately high tensile strengths, low Young’s moduli, and greater elongations at break. Han et al. [25] demonstrated the feasibility of preparing blend films combining HPMC and HPC in various ratios. These films exhibited uniform disintegration properties and sufficient tensile strengths. In general, however, no other detailed studies have combined HPMC and HPC as film-forming blends of ODFs. The potential use of this blend as a film former is described below, along with an evaluation of the effects of polymer ratios.

#### 3.1.1. Observation of the Blend Films

**Figure 1:**
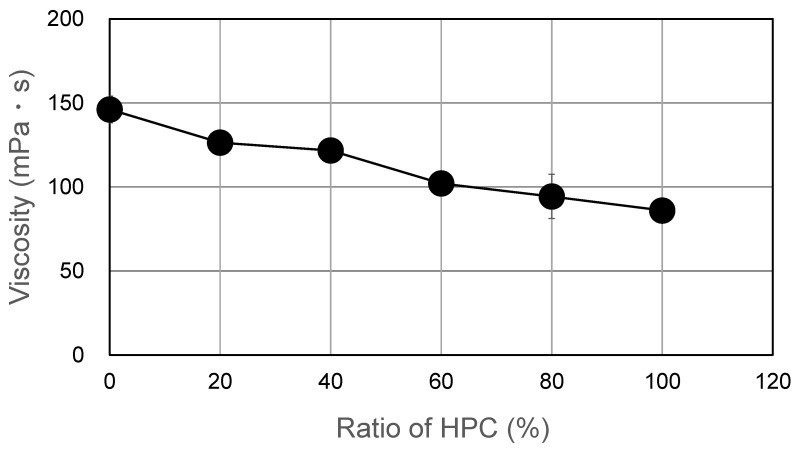
Viscosity of the solution prepared with HPMC/HPC.

**Figure 2:**
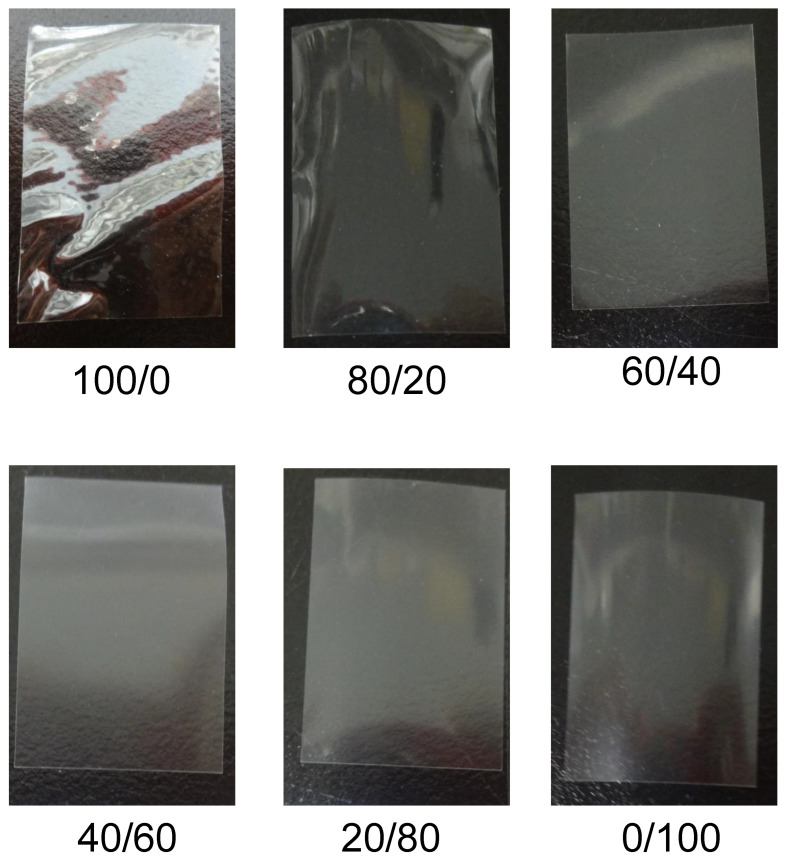
Observation of HPMC/HPC blend films. These films are 20 mm × 30 mm in size.

**Figure 3:**
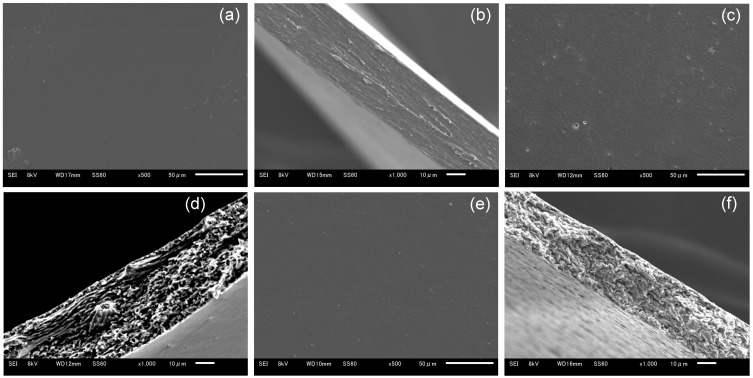
SEM observations of HPMC, HPC, and blend films. (**a**,**b**) HPMC film, (**c**,**d**) HPMC/60% HPC blend film, (**e**,**f**) HPC film. (**a**,**c**,**e**) Surface of films, (**b**,**d**,**f**) cross-sections after tensile test.

Blend polymer solutions formulated with different blend ratios of HPMC/HPC (100/0, 80/20, 60/40, 40/60, 20/80, 0/100) were prepared. Their viscosities are shown in Figure 1. All the films cast from these solutions formed successfully and were peeled from the base surface without any defects. Their morphological images are displayed in Figure 2. All blend films had a desirably smooth appearance, but their transparencies varied based on the HPC ratio. The films with either no HPC or 20% HPC were quite transparent. That with 60% HPC showed the most opacity, though it remained semitransparent. The 100% HPC film was more transparent than the blended films. This observation suggests the macroscopic immiscibility of the blended films, especially for those with around 60% HPC. In addition, using SEM, morphological observations of HPMC film, HPMC/HPC blend films, and HPC film were compared (Figure 3). The scanning electron micrographs of the blend HPMC film with 60% HPC, which were the most semitransparent, are shown here, compared with those of pure HPMC and pure HPC films. Their surfaces were quite smooth (Figure 3a,c,e), while their cross-sections after the tensile test (Figure 3b,d,f) appeared rugged and rough. No clear differences were observed between two pure polymer films and a blend film (HPMC/60% HPC).

#### 3.1.2. Mechanical Properties of the Blend Films

**Figure 4:**
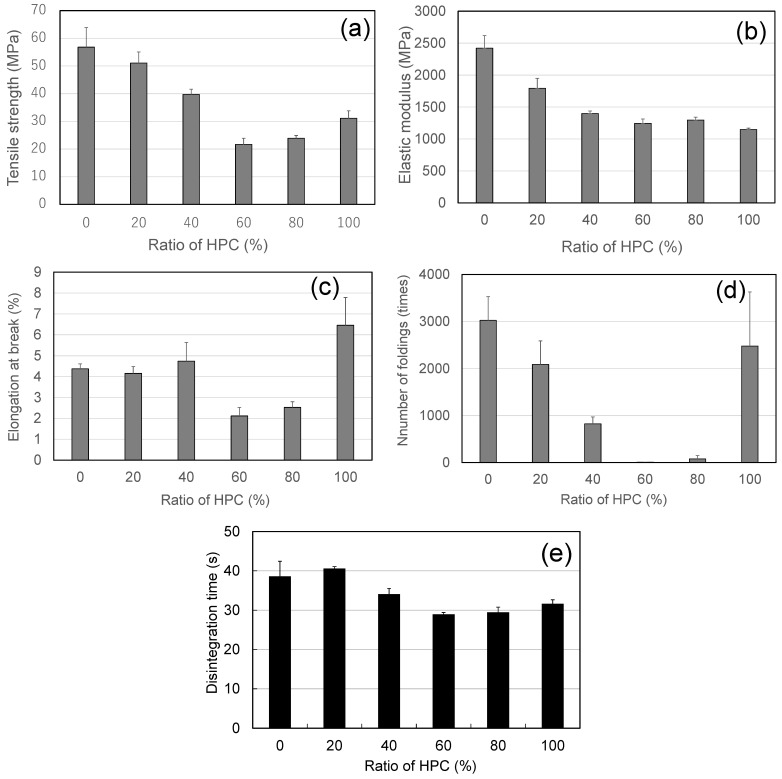
Mechanical and disintegration properties of ODFs prepared from polymer blends with various ratios of HPMC/HPC (standard deviations shown as error bars). (**a**) Tensile strength, (**b**) elastic modulus, (**c**) elongation at break, (**d**) number of foldings, (**e**) disintegration time (using Tricorptester).

Figure 4 shows the mechanical properties of the HPMC/HPC blend films. When the HPC content was 60% or less, the values of TS and EM decreased with the proportion of HPC (Figure 4a–c). These values (TS and EM) for films with 60% and 80% HPC were not significantly different (*p* < 0.05), but they were significantly lower than those of the other films. The EM values for the pure HPC film were the lowest among the films studied (Figure 4b). The FE values also decreased with the increase in HPC; the value was lowest at 60% HPC (about 8 times).

Teramoto et al. [26] reported that the minor component of the blend should be well dispersed in the matrix of the “rich” component, and that the poor dispersion observed in a ~50/50% ratio blend is consistent with the films’ low TS. From this point of view, in the case of the studied HPMC/HPC blend films, HPC behaved as a well-dispersed minor component at ratios below 60% HPC; conversely, at ratios exceeding 80% HPC, HPMC was expected to behave as a well-dispersed minor component. In these cases, the minor-component polymer dispersed successfully in the dominant polymer. However, in the blend films containing 60% or 80% HPC, one of the component polymers could not be uniformly dispersed in the other. Therefore, the HPMC film with 60% and 80% HPC exhibited the lowest TS and the lowest EB among the blend films and showed some opacity rather than being fully transparent. The low TS and EB of blend films with 60% HPC led to the lowest FE value [21].

#### 3.1.3. Disintegration Property

All the blend films formulated here disintegrated in less than 40 s (Figure 4e). DTs of the films with 60% and 80% HPC were significantly (*p* < 0.05) shorter (less than 30 s) than those of the other blend films and the pure HPMC film. The short DTs of these two films also corresponded with the films’ inferior mechanical properties and their morphological cloudiness. These results were attributed to the films’ imperfect, i.e., immiscible, structures.

In conclusion, the 40/60 and 20/80 HPMC/HPC blend films had the shortest DTs of less than 30 s and had the lowest TSs.

### 3.2. Miscibility of the HPMC/HPC Blend Films

The preceding sections showed that films could be formed for any blend ratio of HPMC and HPC. However, morphological observations revealed cloudiness in the blend films with 40%, 60%, and 80% HPC, with the mechanical strength (specifically TS and FE) of the blend film with 60% HPC being the weakest. These results suggested the macro-immiscibility of the polymers in the blend films within the 60–80% HPC range. The miscibility of the blend polymers is usually evaluated through various methods such as morphological observation, mechanical tests, and thermal tests [8]. The next few sections discuss the FTIR and DSC analyses used to study the miscibility of HPMC/HPC blend films.

#### 3.2.1. Differential Scanning Calorimetry

**Figure 5:**
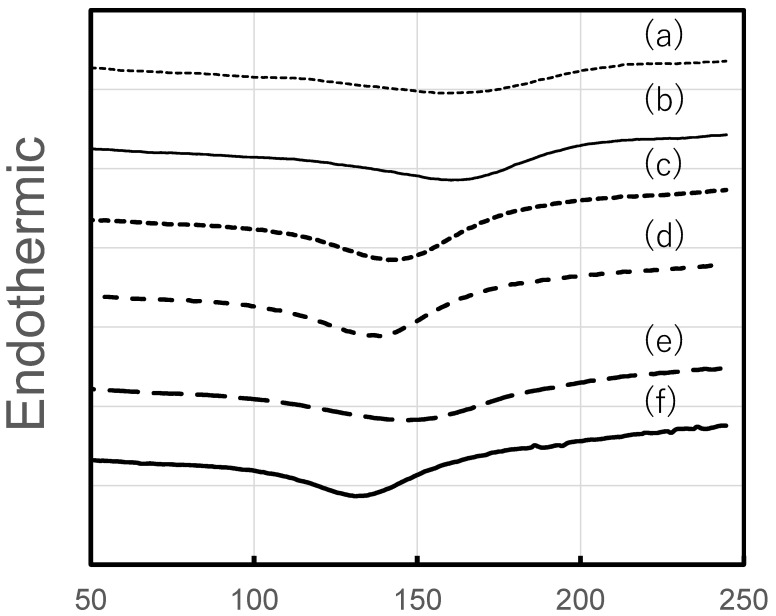
DSC curves of HPMC/HPC blend films. HPMC/HPC ratios (estimated Tg): (a) 100/0 (116 °C), (b) 80/20 (116 °C), (c) 60/40 (110 °C), (d) 40/60 (104 °C), (e) 20/80 (103 °C), (f) 0/100 (102 °C).

DSC curves of the HPMC, HPC, and HPMC/HPC blend films are shown in Figure 5. Endothermic peaks were observed from about 160 °C to 130 °C, and the temperature at the peak decreased with increasing HPC content. Using these curves, the glass transition temperature (Tg) of the blend films was estimated simply as the temperature at the intersection of the two extrapolated lines. The Tg values of the 100% HPMC and 100% HPC films were around 116 °C and around 102 °C, respectively. The Tg values shifted with the addition of HPC, resulting in single value for each blend film. This means that the DSC curves did not show two Tgs, which should theoretically be observed in immiscible blend films. The Tg value of blend films with 20% HPC was around 116 °C, and that of the blend film with 40% HPC was 110 °C. On the other hand, with increased HPC content, the Tg values decreased to around 104 °C and 103 °C (for 60% and 80% HPC, respectively), nearing the value for pure HPC.

Nyamweya and Hoag [8] investigated various blend films with different polymer compositions. For HPMC/HPC blend films (at ratios of 90/10, 75/25, 50/50, 25/75, and 10/90), they found that Tg was not altered by the addition of HPC into HPMC films. Additionally, they observed phase separation caused by the absence of favorable interactions between the two polymers. They concluded that HPMC/HPC blends were immiscible across all compositions. Similarly, our results suggested the immiscibility of these two polymers. This implies the absence of favorable interaction between the two polymers.

#### 3.2.2. Fourier Transform Infrared Spectroscopy

**Figure 6:**
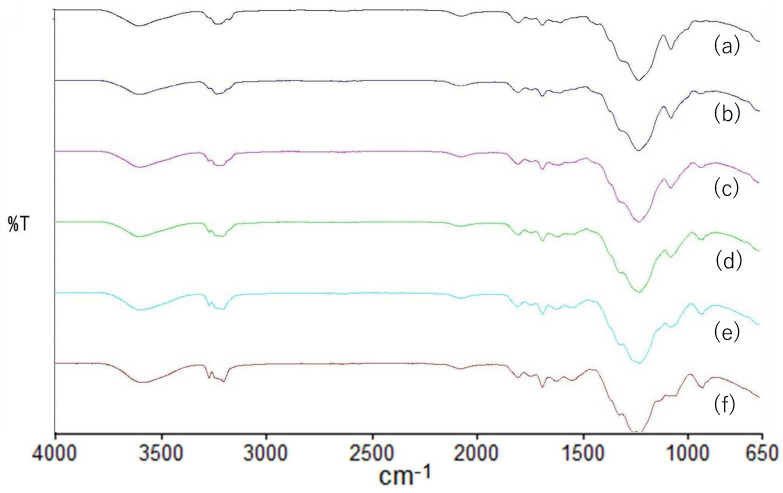
FT-IR spectra of HPMC/HPC blend films. HPMC/HPC ratio: (a) 100/0, (b) 80/20, (c) 60/40, (d) 40/60, (e) 20/80, (f) 0/100.

FTIR spectra of the HPMC, HPC and HPMC/HPC blend films are shown in Figure 6. The HPMC spectra exhibited an absorption band at 3454 cm^−1^, arising from the stretching frequency of the –OH group, along with additional bands at 2899 cm^−1^ (C-H stretching vibration), 1374 cm^−1^ (–OH bending vibration), and 1055 cm^−1^ (C-O stretching vibration). Conversely, the HPC spectra displayed absorption bands at 3422 (–OH stretching), 2971 and 2875 (-C-H stretching vibration, CH_2_ group), 1374 (–OH bending vibration), and 1048 (C-O stretching vibration). The HPMC and HPC peak assignments were based on previous reports, e.g., [27,28]. The spectra of HPMC and HPC showed that these two polymers had very similar structures, except for the –CH_3_ group.

In the spectra of the blend films, the bands at 3454 (cm^−1^) of HPMC films shifted to 3452, 3447, 3448, and 3419 (cm^−1^) for 80/20, 60/40, 40/60, and 20/80 (HPMC/HPC) blends, respectively, while the bands at 1055 (cm^−1^) of HPMC films shifted to 1052, 1051, 1051, and 1049 (cm^−1^) for the various blend films. However, the bands of HPMC films at around 2899 (cm^−1^) (C-H stretching) or around 1454 (cm^−1^) (CH_3_ asymmetric bending vibration) did not shift at all in the blend films. In the observation of the spectra, these peak displacements suggested a certain degree of interaction between the two polymers [29]. However, there were no new peaks indicating chemical interactions [30]. This suggested that one of the polymers was dispersed within the matrix of the other. It was inferred that there were limited and inadequate interactions. These results corroborated the separation of the two polymers, aligning with the morphological observations (Figure 2).

Nyamweya et al. [8] classified the structures of the blend films into three groups—miscible, partially miscible, and immiscible—depending on the nature of the interactions among the individual components. Our results led us to conclude that the HPMC/HPC blend films at all ratios were micro-immiscible, but when the amount of the minor component was sufficiently small, they were macro-miscible, with the minor component dispersing into the major component. As the amount of the minor component increased and exceeded a certain threshold, it could not disperse sufficiently. The blend films containing 60% and 80% HPC exhibited clear phase separation, signifying macro-immiscible structures.

In general, the component polymers in most blended films are micro-immiscible [31]. Nonetheless, there is no practical problem even if micro-phase separation occurs. Even in the case of ODFs, if a blend film can be formed into a thin film, it can be used. Conversely, macro-phase separation resulting from the blend ratio of the polymers can compromise the films’ mechanical strength (TS and FE values) and appearance. For example, a substantial decrease in TS or FE causes practical problems, such as a shortage of APIs and difficulty in handling. In particular, films with substantial API content may experience a further decrease in TS and FE [23]. The present study revealed that the blend films with 60% or 80% HPC were weaker than the 100% HPMC and 100% HPC films. However, these blend films proved to be stronger than many commercially available ODFs. A decrease in disintegration time was also observed. Thus, the blending of HPMC and HPC reduced disintegration time while maintaining sufficient strength.

### 3.3. Characteristics of the HPMC/HPC Blend Films with API

**Figure 7:**
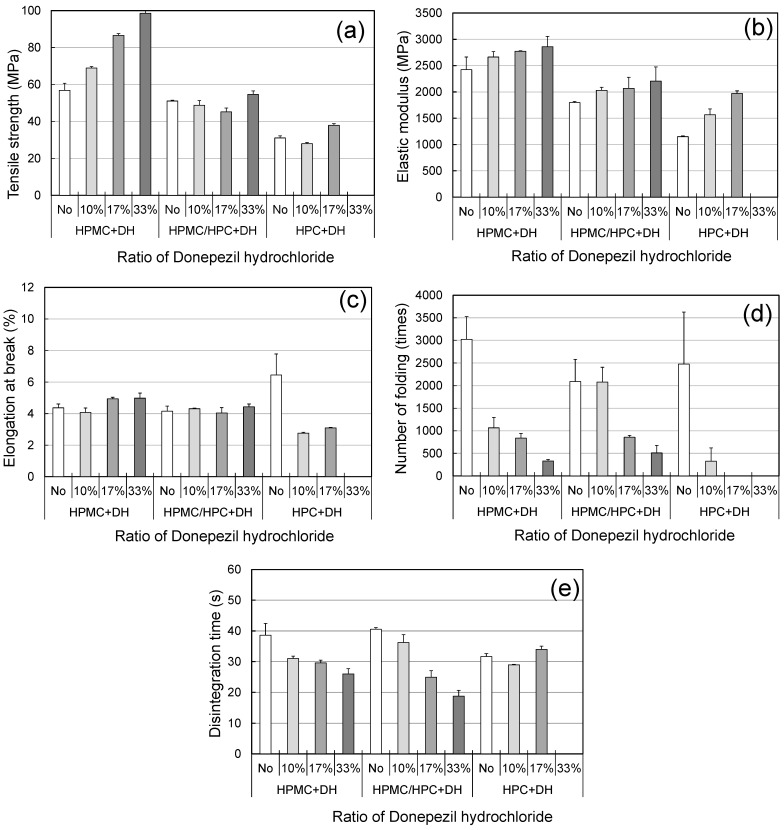
Mechanical and disintegration properties of ODF prepared from polymer blends and DH as a model API (standard deviations shown as error bars). (**a**) Tensile strength, (**b**) elastic modulus, (**c**) elongation at break, (**d**) number of foldings, (**e**) disintegration time (using Tricorptester).

ODFs are required to have sufficiently high mechanical properties, such as high tensile strength and high folding endurance [12,23]. One of the most difficult issues when formulating ODFs loaded with APIs is the degradation of mechanical and disintegration properties due to the addition of APIs. Those properties of the placebo and the loaded films should be compared. To evaluate the feasibility of the HPMC/HPC blend films as film-forming agents for ODFs, we prepared ODFs containing donepezil hydrochloride (DH) as a model API. When evaluating commercially available ODFs, the levels of DH equivalent to 3, 5, and 10 mg per film were calculated (donepezil hydrochloride OD films 3 mg EE, donepezil hydrochloride OD films 5 mg EE, donepezil hydrochloride OD films 10 mg EE: Kyukyu pharmaceutical Co.,LTD.,Tokyo, Japan). The blend films consisted of 80% HPMC and 20% HPC polymers. DH films were successfully produced, with the exception of the HPC film with 33% DH. Figure 7 shows the effect of the DH level on the characteristics of HPMC, HPC, and blend films. Across all films, TS and EM rose with higher DH levels (10%, 17%, and 33%), but EB did not change. The FE values exhibited an extreme decrease as the DH level increased. It was reported that FE was influenced predominantly by both the TS and EB of films [23]. The present results showed that the addition of DH caused the films to become brittle. In the case of the blend film, HPC helped to suppress the increase in TS and the decrease in FE.

**Figure 8:**
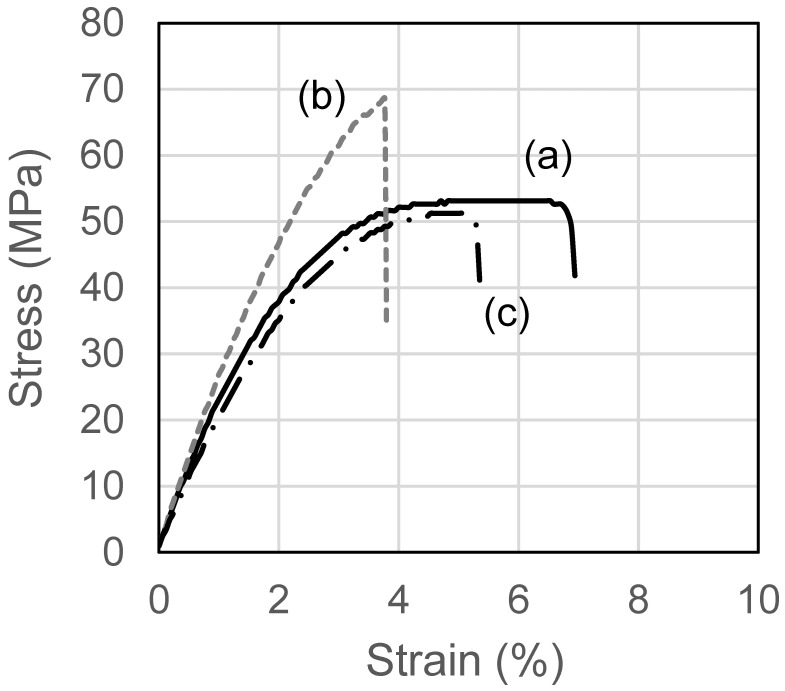
Stress–strain curves of ODFs with DH. (a) HPMC film, (b) HPMC film with DH, (c) HPMC/HPC blend film with DH.

To clarify the advantages of blend films on mechanical properties, the stress–strain curves of these films are illustrated in Figure 8, where, representatively, unloaded HPMC films, HPMC films loaded with 10% DH, and HPMC/HPC blend films loaded with 10% DH are compared. These curves also show that the HPMC film with 10% DH (Figure 8b) fractured more quickly than the other two films, and the introduction of DH rendered HPMC films (Figure 8a) more brittle. The addition of DH to the HPMC/HPC blend film did not change the fracture behavior (Figure 8c), implying that the brittleness effect of DH was suppressed in the HPMC/HPC blend film.

Overall, in this study, the addition of HPC into HPMC film formulations demonstrated positive potential, including a synergistic effect on the inherent properties of pure HPMC.

## 4. Conclusions

The present results verified the feasibility and applicability of HPMC/HPC blend film formers for ODF formulations. (1) HPMC/HPC blends with lower proportions of HPC produced robust ODFs. (2) Disintegration times for the 40/60 and 20/80 ratios of the two polymers were the shortest among the blend films. (3) The HPMC/HPC blend films across all ratios were micro-immiscible, but they became macro-miscible when the amount of the minor component was sufficiently small. (4) The detrimental effects on films caused by the addition of DH were alleviated in the blend film.

Together, these results indicated the capability of combining HPMC and HPC polymers to function as film-forming agents and demonstrated HPC’s viability as a useful blending component in HPMC films. The present findings helped to elucidate the potential applications of these blended polymers in ODFs.

## Figures and Tables

**Table 1:** Formulation of ODFs.

Ingredient	HPMC	HPMC/HPC					HPC	HPMC/DH			HPMC/HPC/DH *			HPC/DH		
HPMC	100.0	90.0	80.0	60.0	40.0	20.0	-	90.0	83.3	66.7	72.0	66.7	53.3	-	-	-
HPC	-	10.0	20.0	40.0	60.0	80.0	100.0	-	-	-	18.0	16.7	13.3	90.0	83.3	66.7
DH	-	-	-	-	-	-	-	10.0	16.7	33.3	10.0	16.7	33.3	10.0	16.7	33.3
Total (%)	100.0	100.0	100.0	100.0	100.0	100.0	100.0	100.0	100.0	100.0	100.0	100.0	100.0	100.0	100.0	100.0

HPMC, hydroxypropyl methylcellulose; HPC, hydroxypropyl cellulose; DH, donepezil hydrochloride. Solvent: ethanol/distilled water = 2:1. Polymer (film former)/solvent = 1:10. * In DH films, HPMC/HPC = 80:20.

## Data Availability

The original contributions presented in this study are included in the article. Further inquiries can be directed to the corresponding authors.
